# Tragic result of traditional Chinese medicine manipulation: an unusual case report of bilateral anterior shoulder dislocations

**DOI:** 10.1186/s12906-015-0633-4

**Published:** 2015-03-31

**Authors:** Chiu-Liang Chen, Shin-Lin Chiu, Chiao-Lee Chu, Shou-Jen Lan

**Affiliations:** Department of Orthopedics, Changhua Christian Hospital, No.135, Nanxiao St., Changhua City, Changhua County 500 Taiwan; Department of Ophthalmology, Changhua Christian Hospital, No.135, Nanxiao St., Changhua City, Changhua County 500 Taiwan; Department of Healthcare Administration, Asia University, No.500, Liufeng Rd., Wufeng Dist., Taichung City, 413 Taiwan

**Keywords:** Bilateral shoulder dislocations, Complementary and alternative medicine, Traditional Chinese medicine, Bonesetter, Complication, Manipulation

## Abstract

**Background:**

In Taiwan, there is a good universal healthcare system to the patients; however, the majority of Taiwanese seek the complementary and alternative medicine when they are injured or ill. The traditional Chinese medicine, which is a branch of complementary alternative medicine, is prevalent in Taiwan. Without proper sequence of maneuvers, either traditional Chinese medicine or conventional medicine might cause unexpected complications. We report a case of 76-year-old woman who was manipulated by a bonesetter, leading to bilateral anterior shoulder dislocations. To the authors’ best knowledge, this injury mechanism of bilateral shoulder dislocations has not been reported in the literature. Since the traditional Chinese medicine is popular in Taiwan, proper training with better skills for the practitioners should be emphasized. We highlight the integration and collaboration of traditional Chinese medicine with western medicine to achieve a better health care for the patients.

**Case presentation:**

A 76-year-old Taiwanese woman has been suffering from soreness and stiffness of bilateral shoulders for 6 months. She went to a bonesetter for mobilization for her shoulders. After manipulations for bilateral shoulders, the woman experienced locked both shoulders with sharp pain. She came to our institute, where the radiographs confirmed bilateral anterior shoulder dislocations. Closed reduction for the dislocations was done by the physician. The patient returned to good range of motion of bilateral shoulders after subsequent rehabilitation without any neurologic deficits.

**Conclusions:**

To the authors’ best knowledge, this unusual injury of bilateral anterior shoulder dislocations had not been reported. The possible mechanism of this injury and the health belief of traditional Chinese medicine in Taiwan are discussed. Improper shoulder manipulations would lead to unexpected complication in any medical practices. We suggest that both traditional Chinese medicine and conventional medicine should follow specific sequences of manipulations; Collaboration and integration with each other could achieve a better healthcare for the patients.

## Background

Taiwan has established a good universal healthcare system for its citizens [1,2]; however, there are a lot of Taiwanese seek the complementary and alternative medicine (CAM) prior to seeking the western medicine. The traditional Chinese medicine (TCM) is a major branch of CAM in Taiwan [3]. Taiwanese are proud of TCM therapy because they think of TCM as a culture prestige of Chinese people and see TCM as a gentle and harmless medicine. The Qigong [4] is an example playing a major role in TCM. Therefore, some unsatisfied patients [5] from conventional therapies refer to TCM treatments.

The TCM has a wide spectrum of therapeutic effects. Either in western medicine or in TCM, without following specific steps, improper manipulations would cause unexpected complications as the case we are showing. We report a case of a 76-year-old woman manipulated by a bonesetter with an unhappy result of bilateral anterior shoulder dislocations. The possible mechanism of bilateral shoulder dislocations and health belief of TCM are discussed.

## Case presentation

**Figure 1:**
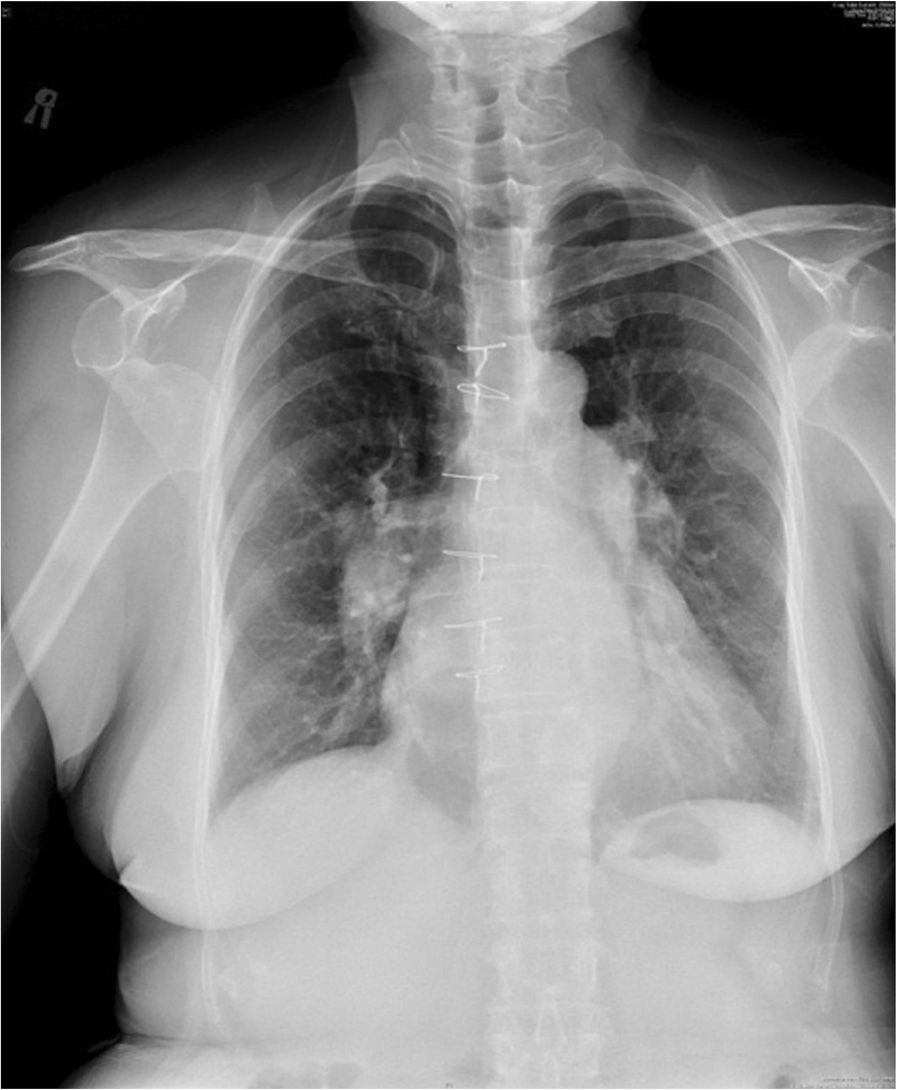
**Chest radiograph demonstrating bilateral anterior shoulder dislocations.**

A 76-year-old Taiwanese woman was presented to the emergency department of our institute with chief complaint of severely painful arms and limitation of movement in both shoulders. Her both shoulders were locked with abduction and internal rotation. She had cardiac surgery 10 years ago. Her past medical history was unremarkable. There were no histories of seizure or epilepsy. She denied drug use or alcohol intake. She had no family history of connective tissue disease, musculoskeletal disorder, or any other seizure disorder. The day before attending our emergent room, she turned to a bonesetter [6,7] for shoulder joint mobilization and manipulation due to chronic soreness and stiffness of her shoulders for 6 months. Without any radiographic or ultrasound examinations, we could not clarify the shoulder pathology before the bonesetter’s manipulation. She had range of motion with abduction of shoulders with 100 degrees. The bonesetter manipulated her right shoulder with abduction and external rotation and a forceful anteriorly push, leading to a snapping sound and a sharp pain over her right shoulder. He explained that the sound meant the Qi flow [8] was patent and it was OK. The patient believed him and received the same manipulation method for the other shoulder subsequently. Her left arm had the same sensation of pain. Her both arms were painful with limited range of motion (Figure 1).

**Figure 2:**
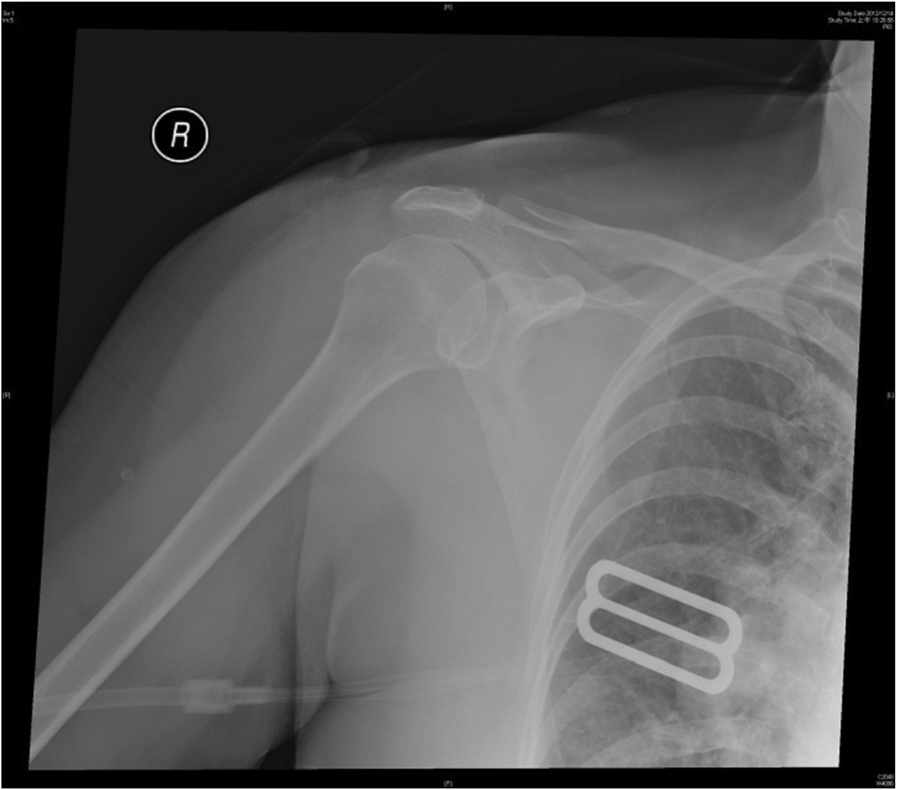
**Right shoulder radiograph showing successful closed reduction.**

**Figure 3:**
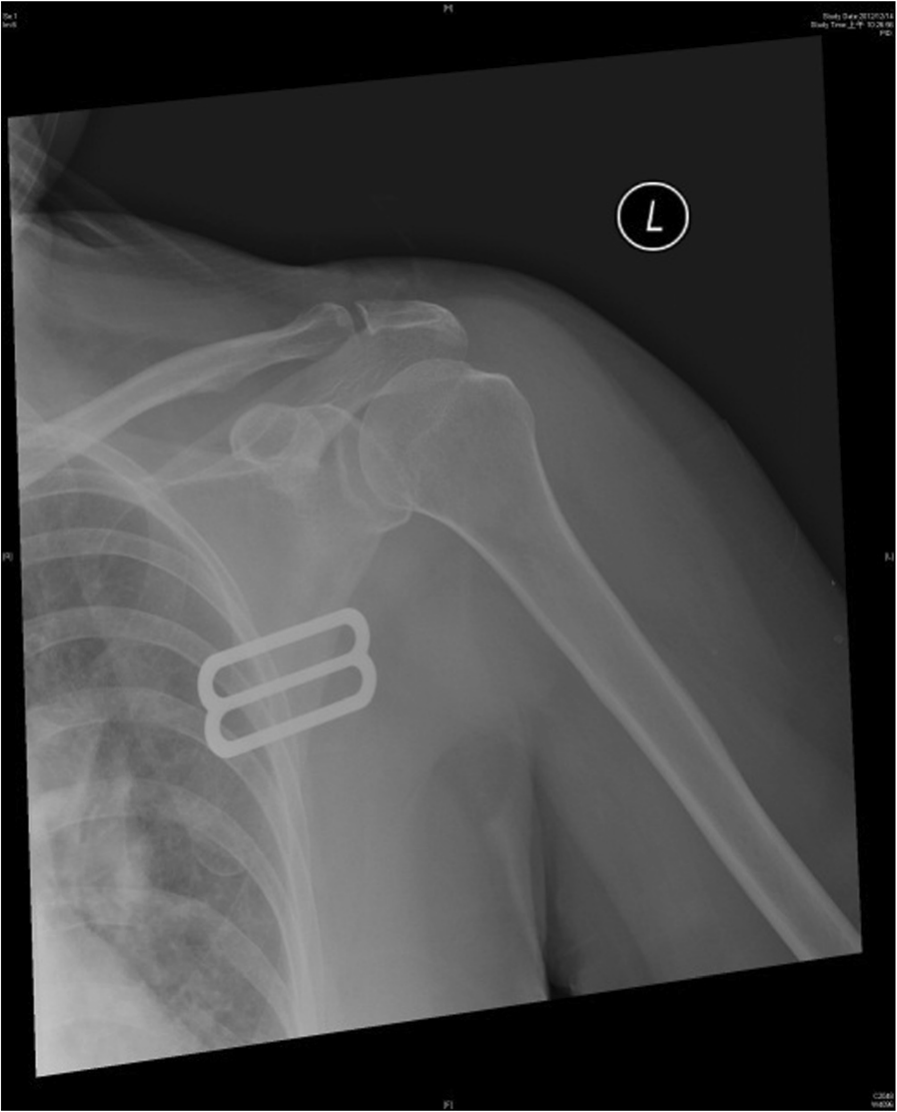
**Left shoulder radiograph showing successful closed reduction.**

The following morning, she still experienced painfully locked shoulders, so she rushed to our institute. On physical examination, the patient’s both upper arms were bruised with hypoesthesia. Both shoulders revealed flattened and squared lateral contour with arms in abduction and external rotation. The radiographs confirmed bilateral anterior shoulder dislocations without fracture. The physician performed closed reduction for her shoulders, using the Kocher’s method [9]. Post-reduction films of shoulders showed successful reduction (Figures 2 and 3). Fortunately, her sensory, motor, and vascular functions were intact after successful reduction. The ultrasonic examination showed no rotators cuff tears. The patient was placed in bilateral slings for 6 weeks with progressive mobilization, starting rehabilitation course at 2 weeks with pendulum movement exercise of both shoulders. Six months after the reduction, the patient recovered to 100 degrees of abduction of both shoulders with little neurovascular deficits.

## Discussion and conclusions

The glenohumeral joint dislocation is the most common type of joint dislocation. Bilateral dislocations of the glenohumeral joint in posterior [10], inferior [11], and anterior [12] directions have been reported. Brown [13] classified bilateral shoulder dislocation in three etiological categories: acute muscular violence, traumatic, and atraumatic. Although anterior shoulder dislocation is the most common major dislocation encountered in the emergent rooms [14], bilateral anterior shoulder dislocations is very rare. Anterior shoulder dislocation, affecting mainly young men(70%, mean age of 33.5 years) and middle age women(mean age of 57.2 years), has a bimodal distribution [12]. The injury mechanism of unilateral anterior shoulder dislocation is forced extension, abduction, and external rotation. However, the mechanism necessary to produce bilateral injury is unusual [14], and is usually of traumatic origin. Other reported cases are caused by seizure, electrocution, hypoglycemic attack, and rheumatoid arthritis [15]. Ballesteros [12] followed Brown’s classification and distinguished different etiologies of anterior shoulder dislocations in subcategories, including traumatic, lever mechanism, traction mechanism, push-on mechanism, unknown or complex mechanism, muscle contraction, and atraumatic.

To our best knowledge, our case is a very unusual case with bilateral anterior shoulder dislocations which has not been reported. The catastrophic complication was produced by a forceful manipulation with shoulder in abduction and external position. The improper joint mobilization or manipulation by the bonesetter led to this tragic result. Medical pluralism is common in Taiwan [16]. The bonesetter [17] is a wide-spectrum term not specific to TCM; however, it plays an important part in TCM in Taiwan. The bonesetters are often regarded as the symbol of TCM practitioners. Although our patient did experience right shoulder pain after joint mobilization by the bonesetter, the pain did not stop her from receiving manipulation of the other shoulder. The possible reason supporting our patient to receive consecutive painful manipulations of both shoulders could be her stronger positive belief in TCM.

CAM is the use of treatments that are not commonly performed by the medical clinicians. As the definition made by the National Center of complementary and alternative medicine, “it is a group of diverse medical and healthcare systems, practices, and products that are not generally considered part of conventional medicine” [18]. The use of CAM are common in both developing countries [19] and industrialized countries with well-organized healthcare system [20,21]. In Australia, sixty percent to eighty percent of patients who visit chiropractic, osteopathic, or Chinese medicine practitioners are seeking pain relief [22]. Nearly forty percent of Americans used CAM [21]. Some patients seek CAM because they are dissatisfied with western medicine that are perceived to be ineffective or have unpleasant side effects [5]. Some patients find CAM attractive because it is inexpensive, culturally similar, and consonant with their health belief and general philosophies [23,24]. Patients want to be in control of their own health, enjoy the personal experience of CAM, and believe CAM can actually help them be healthier and live longer [23].

In Taiwan, CAM is also very prevalent and TCM plays an important role in CAM [25,26]. Though Taiwan has a good national health insurance system [27], many Taiwanese seek TCM or bonesetters when they are sick or injured [26]. It may be for reasons that TCM emphasizes gentle remedies and improving body harmony [23]. Nonetheless, most of the evidences to support the TCM are lacking [25]. Since the CAM and TCM are increasing prevalence in many countries, substantial training and proper manipulation skills need to be emphasized. Unexpected results would happen both in western medicine and TCM if the manipulations are not properly practiced [6]. As the TCM is an important part of CAM with increasingly acceptance worldwide and more potential to be utilized [16,26], the more researches of TCM are mandatory. TCM techniques, as well as the western medicine techniques, should be properly performed to avoid complications. Either TCM or western medicine needs systematic training and proper sequences of maneuvers to decrease unwanted injuries and achieve better results.

In conclusion, our case demonstrated an unhappy result of bilateral anterior shoulder dislocations due to an improper manipulation by a bonesetter. Although shoulder manipulation is widely used by physicians and CAM or TCM practitioners, there is a specific sequence of maneuvers that should be follow otherwise there is a high risk of fractures, dislocation and injury to the rotator cuff. The government in charge of the public health affair should regulate both medical clinicians and TCM practitioners to improve the quality of medical practices and manipulations. Suboptimal techniques of shoulder manipulations might cause unexpected injuries as shown in our case. Since the TCM practice is more popular in Taiwan, we suggest that both TCM and conventional medicine should integrate and collaborate with each other in the same health coverage system to achieve the most benefit for all the patients.

## Consent

Written informed consent was obtained from the patient for the publication of this report.

## Abbreviations

CAM: Complementary and alternative medicine
TCM: Traditional Chinese medicine
